# Supplementary material for: Pore size estimation in axon-mimicking microfibers with diffusion-relaxation MRI
Source: Magn Reson Med. Author manuscript; Available in PMC 2025 Mar 13. (PMC7617479; doi:10.1002/mrm.29991)
Supplement: Supplementary Materials [file EMS203649-supplement-Supplementary_Materials.pdf]

APPENDIX A

The van Gelderen model,[38] which is based on the Gaussian phase distribution approximation, relates the radial diffusivity $D_\perp$ and the radius r as:

$$D_\perp = \frac{2\gamma^2 G^2 r^4}{bD_0} \sum_{m=1}^{\infty} \frac{t_c}{\alpha_m^6(\alpha_m^2-1)} \times \left[2\frac{\alpha_m^2}{t_c}\delta - 2 + 2e^{-\frac{\alpha_m^2\delta}{t_c}} + 2e^{-\frac{\alpha_m^2\Delta}{t_c}} - e^{-\frac{\alpha_m^2(\Delta-\delta)}{t_c}} - e^{-\frac{\alpha_m^2(\Delta+\delta)}{t_c}} \right], \tag{A1}$$

where $D_0 \equiv D_\parallel$ is the free diffusion coefficient, which is equal to the intra-fiber parallel diffusivity when there is no restriction along the principal axes of the cylinders, γ denotes the gyromagnetic ratio, $\delta/\Delta/G$ are the duration/separation/strength of the diffusion gradient, respectively, $t_c = r^2/D_\parallel$, $b = \gamma^2G^2\delta^2(\Delta-\delta/3)$, and α_m are the roots of the derivative of the Bessel function of the first kind of order 1, $J_1'(\alpha_m) = 0$.

In the Neuman limit,[42] that is, $\Delta >> \delta >> r^2/D_\parallel$, Eq. (A1) becomes

$$D_\perp(r) \approx \frac{7}{48}\frac{\gamma^2G^2\delta r^4}{bD_\parallel}. \tag{A2}$$

In this work, we derived a new solution with a less restrictive limit. For $\Delta >> \delta$ and $\delta \in \left(0, \sim r^2/D_\parallel\right)$, Eq. (A1) becomes,

$$\begin{aligned}
D_\perp(r) &\approx \frac{2\gamma^2G^2r^4}{bD_\parallel}\sum_{m=1}^{\infty}\frac{t_c}{\alpha_m^6(\alpha_m^2-1)}\left[2\alpha_m^2\frac{\delta}{t_c} - 2 + 2e^{-\alpha_m^2\frac{\delta}{t_c}}\right] \\
&= \frac{4\gamma^2G^2r^4}{bD_\parallel}\sum_{m=1}^{\infty}\left[\frac{\alpha_m^2\delta}{\alpha_m^6(\alpha_m^2-1)} - \frac{t_c}{\alpha_m^6(\alpha_m^2-1)} + \frac{t_c}{\alpha_m^6(\alpha_m^2-1)}e^{-\alpha_m^2\frac{\delta}{t_c}}\right] \\
&= \frac{4\gamma^2G^2r^4}{bD_\parallel}\left[\sum_{m=1}^{\infty}\frac{\alpha_m^2\delta}{\alpha_m^6(\alpha_m^2-1)} - t_c\sum_{m=1}^{\infty}\frac{1}{\alpha_m^6(\alpha_m^2-1)}\right. \\
&\quad \left. + t_c\sum_{m=1}^{\infty}\frac{1}{\alpha_m^6(\alpha_m^2-1)}e^{-\alpha_m^2\frac{\delta}{t_c}}\right] \\
&= \frac{4\gamma^2G^2r^4}{bD_\parallel}\left[\delta\frac{7}{48\times4} - t_c\frac{7}{48\times4}\times\frac{12}{41} + t_c\frac{7}{48\times4}\times\frac{12}{41}e^{-\alpha_1^2\frac{\delta}{t_c}}\right] \\
&= \frac{4\gamma^2G^2r^4}{bD_\parallel}\left[\delta\frac{7}{48\times4} - t_c\frac{7}{48\times4}\frac{12}{41}\left(1-e^{-\alpha_1^2\frac{\delta}{t_c}}\right)\right] \\
&= \frac{7}{48}\frac{\gamma^2G^2r^4}{bD_\parallel}\left[\delta - t_c\frac{12}{41}\left(1-e^{-\alpha_1^2\frac{\delta}{t_c}}\right)\right] \\
&= \frac{7}{48}\frac{\gamma^2G^2r^4}{bD_\parallel}\left[\delta - \frac{r^2}{D_\parallel}\frac{12}{41}\left(1-e^{-\alpha_1^2\frac{D_\parallel\delta}{r^2}}\right)\right].
\end{aligned} \tag{A3}$$

To derive the previous expression, we used the following approximations:

$$\begin{aligned}
&\sum_{m=1}^{\infty}\frac{\alpha_m^2}{\alpha_m^6(\alpha_m^2-1)} \approx \frac{7}{48\times4}, \\
&\sum_{m=1}^{\infty}\frac{1}{\alpha_m^6(\alpha_m^2-1)}e^{-\alpha_m^2\frac{\delta}{t_c}} \approx \frac{1}{\alpha_1^6(\alpha_1^2-1)}e^{-\alpha_1^2\frac{\delta}{t_c}}, \\
&\sum_{m=1}^{\infty}\frac{1}{\alpha_m^6(\alpha_m^2-1)} \approx \frac{1}{\alpha_1^6(\alpha_1^2-1)} \approx \frac{7}{48\times4}\times\frac{12}{41}.
\end{aligned} \tag{A4}$$

Note that in Eq. (A3), the radial diffusivity is non-negative $D_\perp(r) \geq 0$ for all radii satisfying the condition:

$$1 - \frac{12}{41}\frac{r^2}{D_\parallel\delta}\left(1 - e^{-\alpha_1^2\frac{D_\parallel\delta}{r^2}}\right) \geq 0. \tag{A5}$$

It can be shown that the inequality in Eq. (A5) is valid for all values of r. For instance, in the limit of small r, $\delta >>$

15222594, 2024, 6, Downloaded from https://onlinelibrary.wiley.com/doi/10.1002/mrm.29991 by Welsh Assembly Government, Wiley Online Library on [08/03/2025]. See the Terms and Conditions (https://onlinelibrary.wiley.com/terms-and-conditions) on Wiley Online Library for rules of use; OA articles are governed by the applicable Creative Commons License

t_c, Eq. (A3) becomes equal to Eq. (A2). Moreover, in the limit of large radii, the exponential term in Eq. (A5) can be expanded using the Taylor series, and we obtain

$$1-\frac{12}{41}\frac{r^2}{D_\parallel\delta}\left(1-1+\alpha_1^2\frac{D_\parallel\delta}{r^2}\right)=1-\frac{\alpha_1^2 12}{41}=0.0078, \quad \text{(A6)}$$

fulfilling the inequality $D_\perp(r)\geq 0$

APPENDIX B

The effective radius can be determined using the following derivation:

$$\begin{aligned}\exp\left(-\frac{2\mathrm{TE}\rho_2}{r_{\text{eff-MRI-R}}}\right) &\approx \frac{\int P(r)r^2\exp\left(-\frac{2\mathrm{TE}\rho_2}{r}\right)\mathrm{d}r}{\int P(r)r^2\mathrm{d}r}\\ &\approx \frac{\int P(r)r^2\left(1-\frac{2\mathrm{TE}\rho_2}{r}\right)\mathrm{d}r}{\int P(r)r^2\mathrm{d}r}, \quad \text{for } \frac{2\mathrm{TE}\rho_2}{r} << 1\\ &= 1-\frac{2\mathrm{TE}\rho_2\int P(r)r\mathrm{d}r}{\int P(r)r^2\mathrm{d}r}\\ &\approx \exp\left(-\frac{2\mathrm{TE}\rho_2\int P(r)r\mathrm{d}r}{\int P(r)r^2\mathrm{d}r}\right).\end{aligned} \quad \text{(B1)}$$

Therefore,

$$r_{\text{eff-MRI-R}}\approx\frac{\int P(r)r^2\mathrm{d}r}{\int P(r)r\mathrm{d}r}=\langle r^2\rangle/\langle r\rangle. \quad \text{(B2)}$$

APPENDIX C

Figure C1 shows the spherical mean diffusion signal for various models, including the van Gelderen model

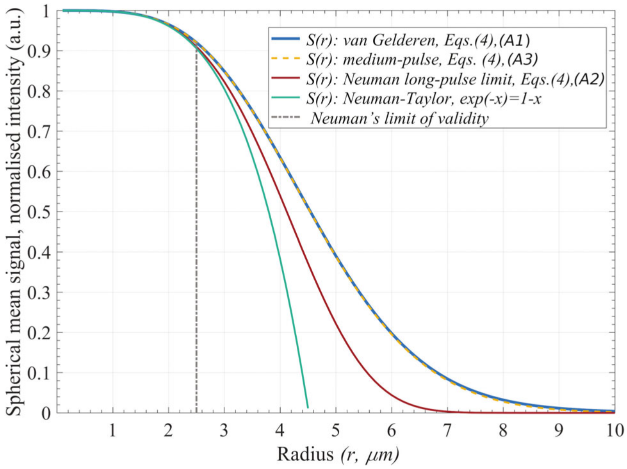

FIGURE C1 Spherical mean diffusion signal as a function of the radius for the acquisition sequence parameters used in this study with $b = 10\,000$ s/mm^2. Four models are displayed, including the van Gelderen model (Eqs. [4] and [A1]), the medium-pulse approximation (Eqs. [4] and [A3]), the Neuman approximation in the long-pulse limit (Eqs. [4] and [A2]) and the first-order Taylor expansion of the Neuman model.

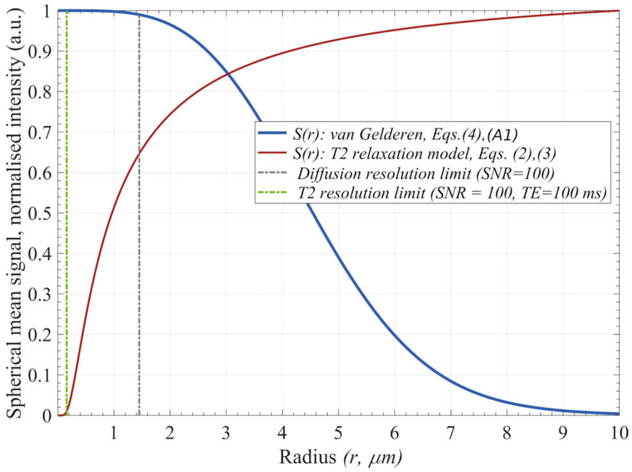

FIGURE C2 Spherical mean diffusion signal and T_2 relaxation signal as a function of the radius for the acquisition sequence parameters used in this study. The diffusion signal was generated for $b = 10\,000$ s/mm^2, and the T_2 relaxation signal was generated for TE = 100 ms, using the parameters $\overline{\rho}_2 = 3.7$ nm/ms and $T_2^b = 3$ s estimated in this study. The resolution limits are shown for the noise level $\sigma = 1/100$, that is, SNR = 100.

(Eqs. [4] and [A1]), the approximation for medium-pulse times (Eqs. [4] and [A3]), the Neuman long-pulse limit (Eqs. [4] and [A2]) and the first-order Taylor approximation of the Neuman model. Note that for fiber radii larger than 2.5 μm, the Neuman approximations deviate from the more accurate van Gelderen model. Conversely, the approximation for medium-pulse times produced accurate results.

Figure C2 displays the spherical mean diffusion and T_2 relaxation signals as a function of the fiber radius for the acquisition parameters used in this study. Moreover, we plot the resolution limits for both normalized signals, defined as the minimum radius for which the signal deviates more than one noise SD σ compared to the signal generated for $r \rightarrow 0$. This definition considers that we cannot accurately detect signal decays smaller than σ. Note that the diffusion resolution limit is >1.4 μm, whereas the T_2-based resolution limit is much smaller, <0.2 μm. The T_2-based resolution limit for shorter TEs is even smaller (result not shown).

APPENDIX D

By considering Eqs. (1), (7), and (8) it is possible to demonstrate that neglecting the relaxation term in the spherical mean power-law approach leads to an effective radius estimate that corresponds to a distorted radius distribution $\widetilde{P}(r)$,

$$\overline{S}_{\text{Diff}}\left(b,\widetilde{r}_{\text{eff-MRI-D}}\right)\approx\frac{\int\widetilde{P}(r)r^2\overline{S}_{\text{Diff}}(b,r)\mathrm{d}r}{\int P(r)r^2\mathrm{d}r}, \quad \text{(D1)}$$

15222594, 2024, 6, Downloaded from https://onlinelibrary.wiley.com/doi/10.1002/mrm.29991 by Welsh Assembly Government, Wiley Online Library on [08/03/2025]. See the Terms and Conditions (https://onlinelibrary.wiley.com/terms-and-conditions) on Wiley Online Library for rules of use; OA articles are governed by the applicable Creative Commons License

where $\widetilde{P}(r) = P(r)\exp\left(-\mathrm{TE}/T_2^i(r)\right)$ is a distorted version of $P(r)$ because of the relaxation process not being modeled in a pure diffusion model. For a constant TE, the signal from the relaxation term $\exp\left(-\mathrm{TE}/T_2^i(r)\right)$ is higher for larger T_2^i times. As T_2^i increases with r, the values of $\widetilde{P}(r)$ for big radii are more inflated than those with small radii. Hence, this approximation leads to overestimating the effective radius calculated by the spherical mean power-law method.

15222594, 2024, 6, Downloaded from https://onlinelibrary.wiley.com/doi/10.1002/mrm.29991 by Welsh Assembly Government, Wiley Online Library on [08/03/2025]. See the Terms and Conditions (https://onlinelibrary.wiley.com/terms-and-conditions) on Wiley Online Library for rules of use; OA articles are governed by the applicable Creative Commons License
